# Better for us all — recent learning on how the Royal College of General Practitioners can reduce racism

**DOI:** 10.3399/bjgpopen20X101150

**Published:** 2020-11-25

**Authors:** Amanda Howe, Martin Marshall, Valerie Vaughan-Dick

**Affiliations:** 1 President, Royal College of General Practitioners, London, UK; 2 Professor of Primary Care, Norwich Medical School, University of East Anglia, Norwich, UK; 3 Chair, Royal College of General Practitioners, London, UK; 4 GP, Liberty Bridge Road Practice, Newham, UK; 5 Professor of Healthcare Improvement, UCL, London, UK; 6 Chief Operating Officer, Royal College of General Practitioners, London, UK

**Keywords:** Equality, Diversity, Inclusivity, General Practice, Primary Health Care

The charitable mission of the Royal College of General Practitioners (RCGP) is to *’* *encourage, foster and maintain the highest possible standards in general medical practice* *‘*, with the implication that this is better for the patients we serve, and for the health of the nation. Within this, there are multiple levels of action typical of professional medical colleges: the curriculum and examination for speciality qualification, ongoing professional development opportunities, guidance on good practice and the latest evidence, membership support, advocacy on behalf of GPs, and many other domains. The College, with its more than 54 000 members, has a wonderfully diverse membership with many different backgrounds, working contexts, and special interests. As a crosscutting effort to ensure we are effectively improving our aim to be an inclusive and diverse organisation in all aspects, we have been updating our Equality Diversity and Inclusivity strategy. We want to embed best organisational practice on an ongoing basis, including when managing resources and staff, as well as responding to members’ needs.

It was in this context that the RCGP had been working with members from Black, Asian, and other minority ethnic groups (‘BAME’) to develop relevant actions, motivated in part by some findings from the *President’s Listening Exercise* conducted during 2018–2019. Members from non-White ethnic backgrounds had raised issues of lack of representation, and the BMA special issue on *Racism* *i* *n Medicine* in February 2020^1^ had begun to trigger action across the sector when the horrific murder of George Floyd in May 2020 shocked the world into action and galvanised the *Black Lives Matter* (BLM) movement. An emergency motion was brought to RCGP Council in June 2020, asking for a clear commitment from the RCGP to the issues raised by the BLM movement, and to tackle structural racism across society, including within general practice and, indeed, with the implication to look at RCGP itself. Questions within RCGP had also been raised about why an apparently democratic electoral process was resulting in an atypical demographic profile in senior RCGP leadership, both in relation to the UK population per se,^2^ and in even greater contrast with the current RCGP membership profile. This has led us to look in more depth at how to move strategic aims into effective action.

So this article focuses on the question: what can and should a professional body do to address such issues?^3^ There is a substantive body of literature derived both from academic studies and the development of good organisational practice,^4^ which advocates for a combination of routine training, data monitoring, and encouraging a shift in organisational awareness and cultural practices. Other principles include active outreach and positive action programmes, which can be defined as ’*a range of measures allowed under the Equality Act 2010 which can be lawfully taken to encourage and train people from under-represented groups to help them overcome disadvantages in competing with other applicants*‘.^5^ Strategies include organisations using relevant images and role models, and — to address and avoid more negative experiences — training in ‘speaking up’, ‘unconscious bias’, or ‘active bystander’ approaches.^6^ Finally, the principles of equity and justice need to be embraced and embedded in relevant organisational practices at every level.

The RCGP has formed five task groups comprised of interested members from diverse backgrounds working under the domains of awareness raising; education and training; professional development; working with external stakeholders; and representation and inclusion. Each of these groups has a remit to consider relevant areas of organisational activity and have come back with recommendations about what could make a difference in the short and medium term. These include review of the MRCGP (Membership of the Royal College of General Practitioners) curriculum; designing training modules for practice teams to address racism in their settings; changing the media images on the RCGP website; collecting member stories for increasing awareness; working with the BMA and other Royal Colleges; continuing our work on reviewing how to address differential attainment and give support for trainees from different backgrounds; and looking specifically about how we can proactively get diversity in all our committees that reflects our membership demographic profile. Ongoing developments on formal representation and measurable targets including in faculty and Council structures are part of this process. We are also continuing to gather the evidence on the altered health risk profile for those of a BAME background, including addressing the worrying recent findings regarding COVID risk,^^7^^ and working hard to ensure these are addressed at practice level, both for staff and their patients. Even the words we use are for debate; there have been calls by members to remove the term ‘BAME’ from our literature and vocabulary, and instead represent our diversity in a more sensitive and meaningful manner. We have taken on this feedback and are consulting on this matter.

Within the last month we have seen real evidence of the difference that the current debate has made, with a much more diverse field of applicants for recent Council elections, and also in the profile of those who were elected. What are the challenges? We are in the midst of a pandemic, and the stressors and risks within general practice are very real. Resources are limited, especially in the current climate. There are other priorities: workforce; workload; retaining the best of relationship-based care while embracing the opportunities of the new digital technologies; and learning how best to work at scale across practice boundaries, while sustaining effective multidisciplinary primary care teams.^8^ Some have raised the need to recognise that prejudice can affect people with other protected characteristics: gender, disability, and sexuality can all become a focus for negativity and can undermine GPs in their daily practice and careers. The challenge of intersectionality, when multiple domains combine to add disadvantage, is even greater. But we are confident that, by holding up a lens to learning about racism and how to prevent it, all areas of the RCGP will become more skilful in practising measures that are inclusive, increase diversity, provide a sense of belonging, and achieve equitable outcomes for all. We are grateful to all the colleagues who have challenged and championed these issues in recent times, and are delighted that *BJGP* *Open* is taking its turn to put the issues centre-stage. This will be better for us all.
